# Three new species of *Misionella* from northern Brazil (Araneae, Haplogynae, Filistatidae)

**DOI:** 10.3897/zookeys.589.7951

**Published:** 2016-05-16

**Authors:** Antonio D. Brescovit, Ivan L. F. Magalhaes, Igor Cizauskas

**Affiliations:** 1 Laboratório Especial de Coleções Zoológicas, Instituto Butantan, Av. Vital Brasil, 1500, 05503-900, São Paulo, SP, Brasil; 2 División Aracnología, Museo Argentino de Ciencias Naturales “Bernardino Rivadavia”, Av. Angel Gallardo 470, C1405DJR, Buenos Aires, Argentina

**Keywords:** Caves, Caatinga, endemics, Prithinae, spiders, taxonomy

## Abstract

Three new species of the genus *Misionella* are described from Brazil: *Misionella carajas* **sp. n.** and *Misionella aikewara* **sp. n.** from caves in the states of Pará and Tocantins and *Misionella pallida* **sp. n.** from natural and synanthropic dry areas in the states of Piauí, Maranhão, Rio Grande do Norte and Bahia. These species seem to belong to a distinct group within the genus; the males have an elongate palpal tibia and bulb, a pair of characteristic and hirsute macrosetae in the second metatarsus and the females have internal genitalia with only one pair of spermathecae, with relatively short ducts, lacking the auxiliary receptacles. Their phylogenetic placement and geographic distribution are briefly discussed.

## Introduction

The genus *Misionella* Ramírez & Grismado, 1997 was established to include the species *Filistata mendensis* described by Mello-Leitão (1920) from Mendes, state of Rio de Janeiro, Brazil. This species is very common in southeastern Brazil and northeastern Argentina, where populations have synanthropic habits, living in the brick walls in houses or on tree bark near buildings or urban squares. Grismado and Ramírez (2000) described *Misionella jaminawa*, a second species from the state of Acre, in Brazilian Amazonia.

**Figure 1.**
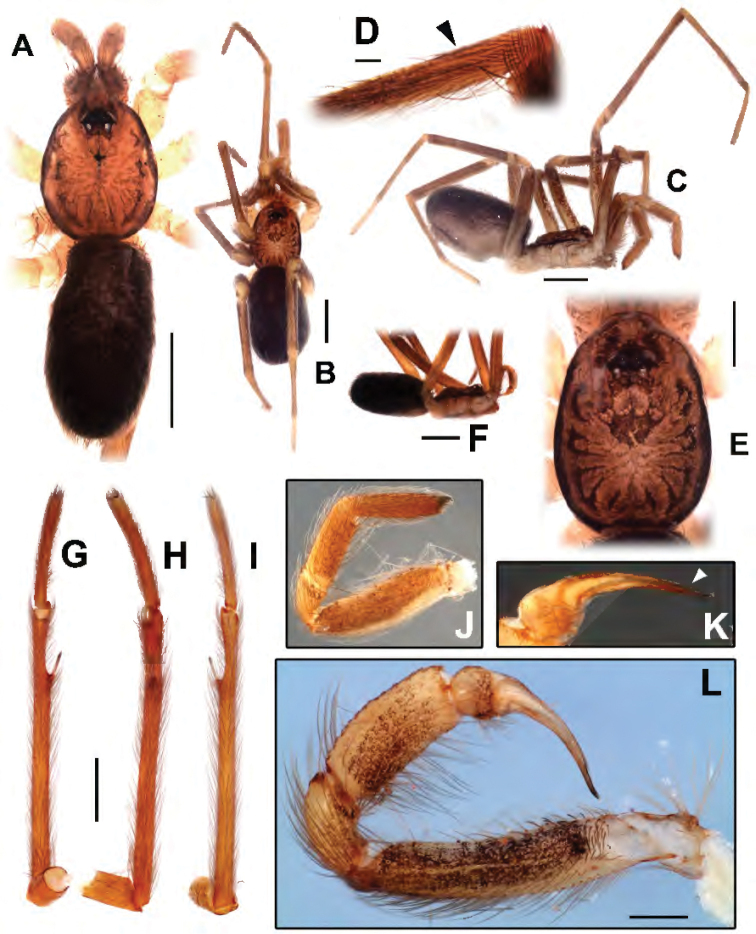
Female from Cave N4E_0079, Flona Carajás, Parauapebas, Pará (IBSP 166200) (**B–E, J**) male from the same locality (IBSP 166199) (**A, F–I, K-L**). **A–B** habitus, dorsal view **C** habitus, lateral view **D** left metatarsus IV retrolateral showing calamistrum (indicated by an arrow) **E** carapace, dorsal view **F** habitus, lateral view **G** left metatarsus II, ventral view **H** same, retrolateral view **I** same, dorsal view **J** right pedipalp **K** left palp, prolateral view, white arrow indicates the paraembolic lamina **L** same, complete male palp, prolateral view. Scale bars: 1 mm, except **G–I** (0.5 mm) and **D, L** (0.1 mm).

**Figure 2.**
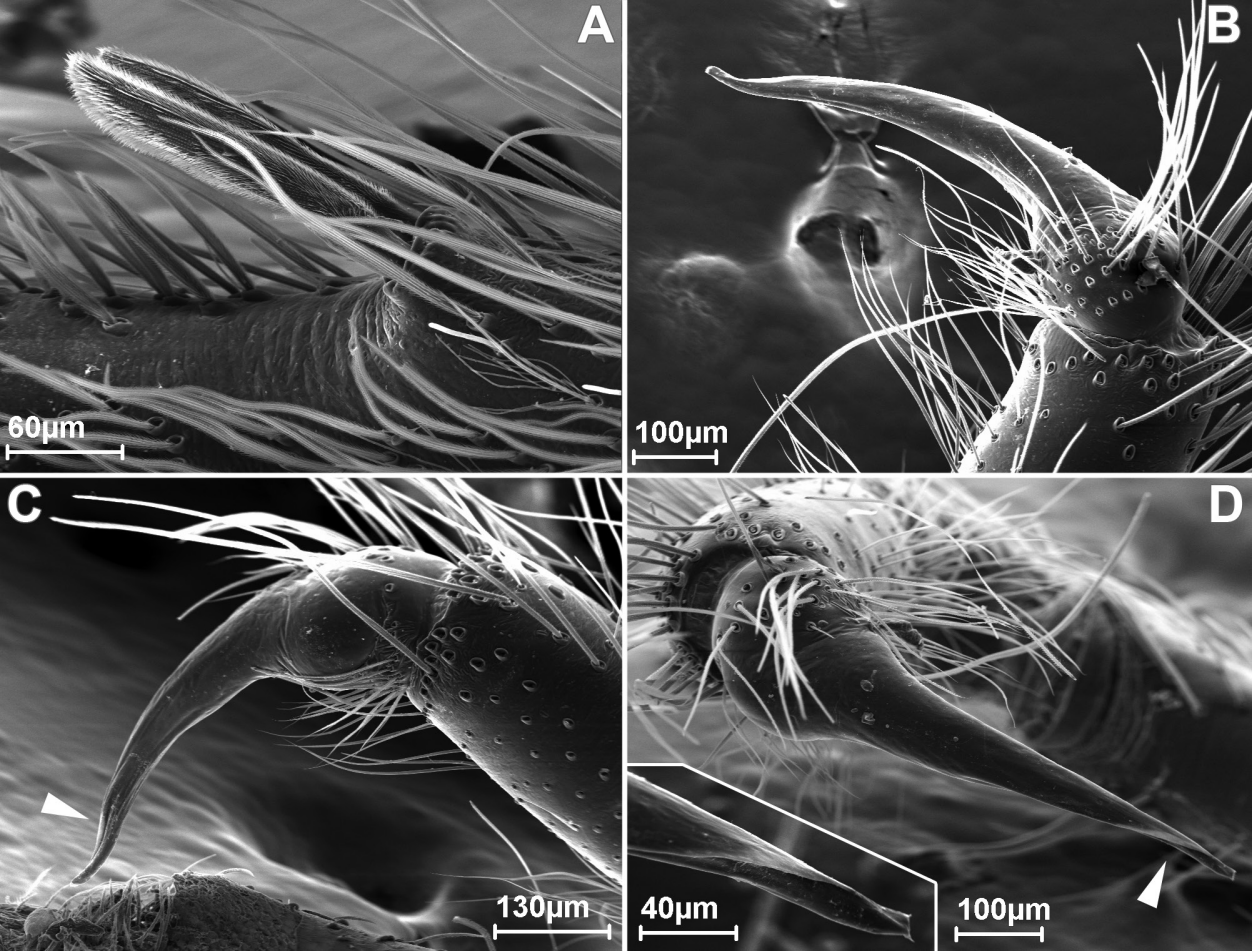
SEM images of *Misionella carajas* sp. n., male from Cave N4E-0020, Flona Carajás, Parauapebas, Pará (IBSP 97841) (**A–D**), **A** male metatarsus II, retrolateral view **B–C** male left palp, prolateral **D** same, dorsal view (inset: detail of embolus tip). White arrows indicate the paraembolic lamina.

The species of the genus *Misionella* resemble those of *Pikelinia* Mello-Leitão in having enlarged male palpal tibia and second male metatarsi retrolaterally excavated, with short spinules (Figs 1–2) or with excavation absent, but having at least a pair of macrosetae (Fig. 1H). According to Ramírez and Grismado (2000) species of these genera differ by the absence of any projection in the male palpal tibia (Ramírez and Grismado 1997, figs 100–102) and by having two pairs of spermathecae placed side by side in the internal female genitalia (see Ramírez and Grismado 1997, Fig. 103) in Misionella. Following the original diagnosis presented by Ramírez and Grismado (2000) the species here described seem to belong to a distinct group in the genus Misionella. The males of these species lack an apophysis on the palpal tibia, have a characteristic pair of hirsute spines in the second metatarsus (Figs 1G–I) and the females have internal genitalia with only one pair of spermathecae with short ducts (Figs 8C, 11C).

In this paper, we describe these three new, morphologically deviant species of *Misionella* from Brazil, two from caves in the states of Pará and Tocantins and a third from dry areas in the states of Piauí, Maranhão, Rio Grande do Norte and Bahia.

## Material and methods

The material examined belongs to the following institutions:

CHNUFPI
 Coleção de História Natural, Universidade Federal do Piauí, Floriano (L.S. Carvalho) 


IBSP
 Instituto Butantan, São Paulo (A.D. Brescovit) 


ISLA
 Zoology Collection, Seção de Invertebrados Subterrâneos da Universidade Federal de Lavras, Lavras (R.L. Ferreira) 


MPEG
Museu Paraense Emílio Goeldi, Belém (A.B. Bonaldo) 


MZSP
 Museu de Zoologia da Universidade de São Paulo, São Paulo (R. Pinto da Rocha) 


UFMG
 Coleções Taxonômicas da Universidade Federal de Minas Gerais, Belo Horizonte (A.J. Santos) 


Descriptions follow Ramírez and Grismado (1997). Measurements are expressed in millimeters. Leg segment lengths were measured laterally. The illustrations were made in a stereomicroscope with *camera lucida*. For the illustration of female genitalia, we used dissected organs immersed in clove oil, following Levi (1965). Image stacks were obtained with a Leica M165 C stereoscopic microscope and extended focus images were generated using Helicon Focus 6 (www.heliconsoft.com); female spermathecae were digested with pancreatin and photographed in temporary mounts without a coverslip in lactic acid on an Olympus BH-2 compound microscope. Material for SEM was either air-dried or dehydrated using an ethanol series followed by immersion in HDMS, and sputter-coated with 10 nm of gold or gold-palladium. Micrographs were taken with a Quanta 250 the electron scanning microscope at Laboratório de Biologia Celular Instituto Butantan, LEO 1450VP of the Museu Paraense Emílio Goeldi, or a Philips FEI XL30 TMP at Museo Argentino de Ciencias Naturales “Bernardino Rivadavia”.

## Taxonomy

### Family Filistatidae Ausserer, 1867 Subfamily Prithinae Gray, 1995

#### Misionella

Taxon classificationAnimaliaAraneaeFilistatidae

Ramírez & Grismado, 1997

Misionella Ramírez & Grismado, 1997: 342, type species Misionella mendensis (Mello-Leitão, 1920), by monotypy and original designation.

##### Diagnosis.

Males of *Misionella* have the cymbium fused to the tegulum, as do *Pikelinia* and *Lihuelistata*; they can be distinguished from *Pikelinia* by the lack of an apophysis on the palpal tibia, and from *Lihuelistata* by the modified second metatarsi (Ramírez and Grismado 1997). Females of *Misionella* can be distinguished from *Kukulcania* Lehtinen by having a three-rowed calamistrum, from *Pikelinia* by having spermathecae either paired side by side or unilobulate, and from *Lihuelistata* by lacking pores on the ducts of the spermathecae (Ramírez and Grismado 1997).

#### Misionella carajas sp. n.

Taxon classificationAnimaliaAraneaeFilistatidae

http://zoobank.org/37090EE0-9A6D-4A39-8E2D-5F79135D97B9

**Figure 3.**
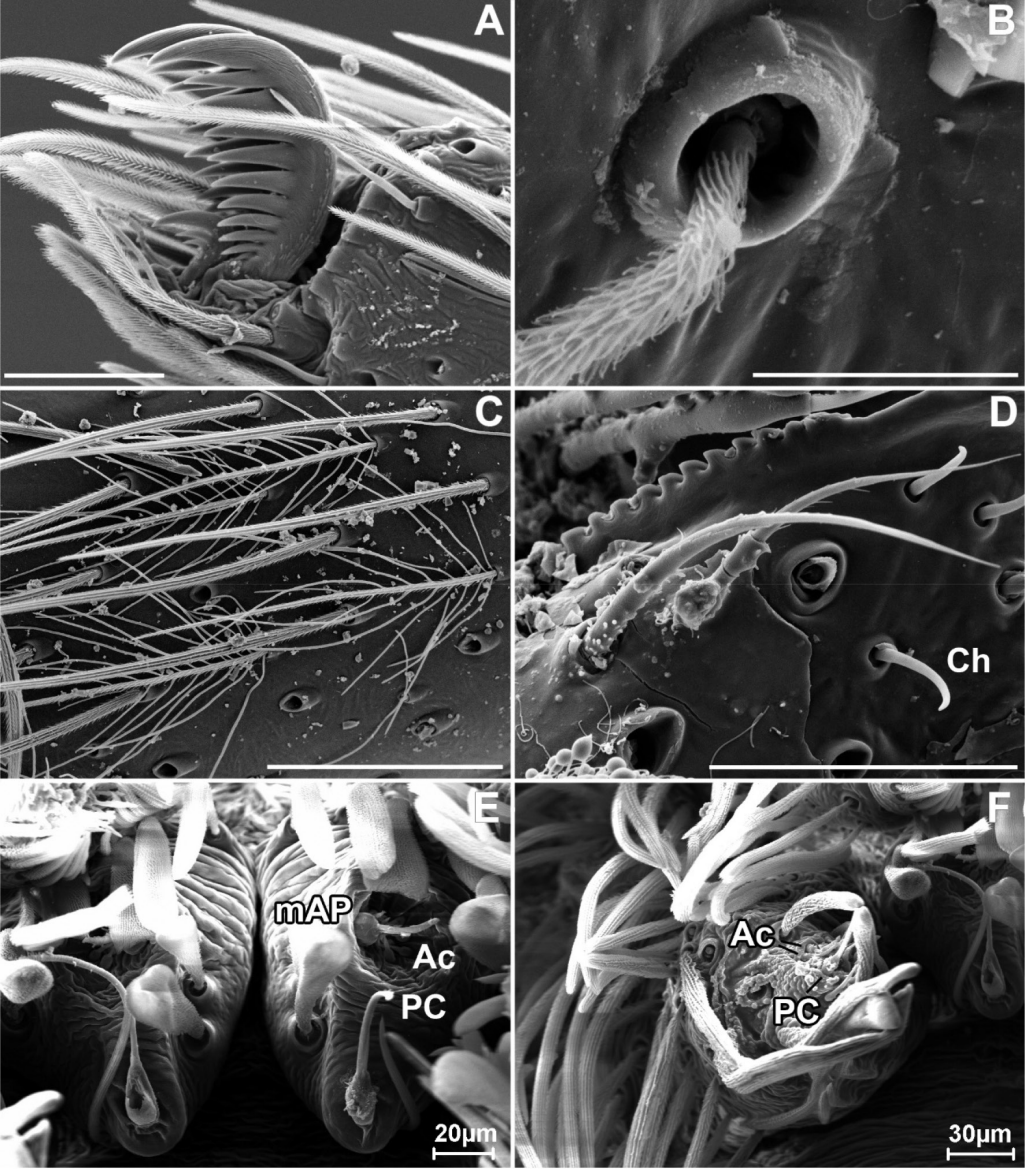
SEM images of *Misionella carajas* sp. n., female from Cave N4E-0024, Flona Carajás, Parauapebas, Pará (IBSP 97831) **A** female right leg I, tarsal claws **B** female right leg III, tarsus, dorsal, trichobothria **C** same, retrolateral, detail of plumose setae **D** endite, serrula and chemosensory setae **E** PMS, ventral view **F** PLS, ventral view. Abbreviations: Ac = aciniform gland spigots, Ch= chemosensory setae, mAP = minor ampullate gland spigot, PC = paracribellar gland spigot. Scale bars: 0.05 mm (**A, D**), 0.01 mm (**B**), 0.1 mm (**C**).

Figs 1A‒L , 2A‒D , 3A‒F , 11A‒C , 12A , 13A‒D , 14

##### Types.

Male holotype from Cave N4E_0024 (06°02'01"S, 50°10'07"W), FLONA Carajás, Parauapebas, Pará, Brazil, 20/IV‒04/V/10, D. B. Pedroso col. (IBSP 161396). Paratypes: female paratype from Cave N5S_0059 (06°06'29"S, 50°07'57"W), FLONA Carajás, Parauapebas, Pará, Brazil, 25/VIII‒03/IX/2009, I. Cizauskas col. (IBSP 161395); two males and two females from Cave N4E_0070 (06°01'56"S, 50°09'10"W), 24-30/VII/2009, I. Cizauskas (MPEG 24927); two males and two females from Cave N4E_0079 (06°01'59"S, 50°09'05"W), 24/VII/2009‒04/III/2010, I. Cizauskas, D.B. Pedroso, J.B. Verdiani & J. Mascarenhas (MZSP 68015)

##### Additional material examined.

BRAZIL. *Pará*: Parauapebas, Flona Carajás, Cave GEM-1797 (06°06'25"S, 50°08'07"W), 3♂ 3♀, 23/VIII/2010, R. Zampaulo col. (IBSP 165001); Cave N1_0002 (06°02'24"S, 50°16'12"W), 2♀, 11/VI–02/VII/2014 (IBSP 181113; 191159); Cave N1_0023 (06°01'13"S, 50°16'40"W), 2♂, 11/VI–02/VII/2014 (IBSP 181114; 191160); Cave N1_0059 (06°01'11"S, 50°16'44"W), 2♂ 2♀, 11/VI-02/VII/2014 (IBSP 181115–181116, 191161‒191162); Cave N1_0063 (06°01'07"S, 50°16'45"W), 4♂ 5♀, 11/VI–02/VII/2014 (IBSP 181117–18119; 191163–191165); Cave N1_0075 (06°01'14"S, 50°16'49"W), 2♂ 1♀, 11/VI–02/VII/2014 (IBSP 181120; 191166); Cave N1_0077 (06°01'14"S, 50°16'52"W), 2♀, 11/VI–02/VII/2014 (IBSP 191167–191168); Cave N1_0080 (06°01'11"S, 50°16'48"W), 2♀, 11/VI–-02/VII/2014 (IBSP 191169); Cave N1_0081 (06°01'13"S, 50°16'47"W), 1♀, 11/VI–02/VII/2014 (IBSP 191170); Cave N1_0083 (06°01'20"S, 50°16'47"W), 1♂ 1♀, 11/VI–02/VII/2014 (IBSP 191171); Cave N1_0087 (06°01'09"S, 50°16'59"W), 1♂ 1♀, 11/VI–02/VII/2014 (IBSP 191172); Cave N1_0094 (06°01'11"S, 50°16'57"W), 2♂ 3♀, 11/VI–02/VII/2014 (IBSP 191173‒191174); Cave N1_0096 (06°01'09"S 50°16'59"W), 1♀, 11/VI–02/VII/2014 (IBSP 191175); Cave N1_0098 (06°01'09"S, 50°17'05"W), 2♀ 2 imm, 28/X–03/XI/2007 (IBSP 97883); Cave N1_0123 (06°01'09"S, 50°16'45"W), 1♀, 11/VI–02/VII/2014 (IBSP 191176); Cave N1_0185 (06°02'35"S, 50°16'33"W), 1♂ 1♀, 16/VII–06/VIII/2014 (IBSP 191187); Cave N1_0224 (06°01'16"S 50°16'19"W), 3♂ 3♀, 11/VI–02/VII/2014 (IBSP 191178-191181); Cave N1_0234 (06°01'15"S, 50°16'24"W), 2♀, 11/VI–02/VII/2014 (IBSP 191182-191183); Cave N1_0236 (06°01'15"S, 50°16'25"W), 1♂, 11/VI–02/VII/2014 (IBSP 191184); Cave N1_0239 (06°01'19"S, 50°16'26"W), 2♀, 11/VI–02/VII/2014 (IBSP 191185–191186); Cave N2_0002 (06°03'13"S 50°14'32"W), 1♂ 3 imm, 26/IX–17/X/2012 (IBSP 191200); Cave N3_0031 (06°02'38"S, 50°13'11"W), 2♀, 03–17/IV/2013 (IBSP 191201); Cave N3_0053 (06°02'27"S, 50°13'45"W), 2♀, 02–23/VIII/2013 (IBSP 191202); Cave N3_0054 (06°02'27"S 50°13'43"W), 1♂, 02–23/VIII/2013 (IBSP 191203); Cave N3_0063 (06°02'33"S, 50°13'37"W), 1♀, 02–23/VIII/2013 (IBSP 191204); Cave N3_0066 (06°02'32"S, 50°13'36"W), 2♂, 02–23/VIII/2013 (IBSP 191205); Cave N3_0069 (06°02'30"S, 50°13'37"W), 2♀, 02–23/VIII/2013 (IBSP 191206), all collected by Equipe Carste; Cave N4E_0004 (06°02'26"S, 50°09'39"W), 2 imm, 20/X–01/XI/2006 (IBSP 97837); Cave N4E_0009 (06°02'21"S, 50°09'36"W), 1♀ 1 imm, 20/X–01/XI/2006 (IBSP 97855); Cave N4E_0012 (06°02'16"S, 50°09'37"W), 1♀ 2 imm, 20/X–01/XI/2006 (IBSP 97836), Cave N4E_0013 (06°02'18"S, 50°09'38"W), 1♀, 20/X–01/XI/2006 (IBSP 97848), all collected by R. Andrade et al.; Cave N4E_0015 (06°02'10"S, 50°09'35"W), 1♀, 20/X–01/XI/2006 R. Andrade (IBSP 97854); 1♀, 20/IV–04/V/10, J. Mascarenhas (IBSP 191142); Cave N4E_0020 (06°02'02"S, 50°09'35"W), 2♂ 1♀ 4 imm, 20/X–01/XI/2006, R. Andrade & I. Cizauskas (IBSP 97841, SEM; IBSP 191157); Cave N4E_0022 (06°02'02"S, 50°10'04"W), 2♀, 20/X–01/XI/2006, R. Andrade et al. (IBSP 97852); Cave N4E_0024 (06°02'01"S, 50°10'07"W), 10♀ 5 imm, 20/IV–04/V/10, D. B. Pedroso, R. Andrade & I. Cizauskas col. (IBSP 97831, SEM; IBSP 161036, IBSP 191143); Cave N4E_0026 (06°02'14"S, 50°10'03"W), 2♂ 4♀ 1 imm, 18/VIII‒03/IX/2009, I. Cizauskas & J. Mascarenhas col. (IBSP 191127–191128; IBSP 191151, MPEG 24925); Cave N4E_0031 (06°02'24"S, 50°09'39"W), 1♀, 08–12/II/2007, R. Andrade et al. (IBSP 97748); Cave N4E_0035 (06°02'19"S, 50°09'38"W), 1♂, 18/VIII–03/IX/2009, J. B. Verdiani (IBSP 191152); Cave N4E_0036 (06°02'08"S, 50°09'36"W), 1 imm., 08–12/II/2007, R. Andrade et al. (IBSP 97740); Cave N4E_0037 (06°02'07"S, 50°09'37"W), 2♀, 08–12/II/2007, R. Andrade et al. (IBSP 97754); Cave N4E_0038 (06°02'05"S, 50°09'37"W), 4♂ 10♀ 10 imm., 08‒12/II/2007, R. Andrade, D. Bebiano & J. B. Verdiani (IBSP 97762; IBSP 191130–191131); Cave N4E_0045 (06°02'25"S, 50°09'40"W), 2♂ 1♀, 1 imm., 19/II–04/III/2010, I. Cizauskas (MPEG 24926; IBSP 191154–191155); Cave N4E_0049 (06°02'14"S, 50°09'36"W), 1♂, 3 imm., 18/VIII–03/IX/2009, D. Bebiano (IBSP 191153); Cave N4E_0050 (06°02'09"S, 50°09'36"W), 1♂, 1♀ 1 imm., 18/VIII‒03/IX/2009, D. B. Pedroso (IBSP 191132); Cave N4E_0052 (06°02'02"S, 50°09'37"W), 2♂ 1♀, 24–30/VII/2009, R. Andrade (IBSP 191133); Cave N4E_0053 (06°02'03"S, 50°10'02"W), 1♂, 1 imm., 24–30/VII/2009, D. Bebiano (IBSP 191156); Cave N4E_0056 (06°01'58"S, 50°09'41"W), 1♀ 3 imm., 19/II–04/III/2010, C.A.R. Souza (IBSP 191135); Cave N4E_0063 (06°02'01"S, 50°09'15"W), 1♀, 24–30/VII/09, J. Mascarenhas (MZSP 68013); Cave N4E_0066 (06°01'52"S, 50°09'03"W), 3♂ 2♀, 17 imm., 24-30/VII/09–19/II-04/III/2010, I. Cizauskas, D.B. Pedroso & J.B. Verdiani (IBSP 191116; IBSP 191136; IBSP 191147–191149); Cave N4E_0079 (06°01'59"S, 50°09'05"W), 14♂ 20♀ 19 imm., 24/VII/2009–04/III/2010, I. Cizauskas, D.B. Pedroso, J.B. Verdiani & J. Mascarenhas (IBSP 166199–166200, IBSP 191119, IBSP 191121–191123, IBSP 191137–191140, MZSP 68014); Cave N4E_0092 (06°02'22"S, 50°09'31"W), 5♂ 3♀ 2 imm., 24‒30/VII/2009, D. B. Pedroso R. Andrade C. A. R. Souza & J. B. Verdiani (IBSP 191125–191126; IBSP 191141; IBSP 191150); Cave N4WS_0006 (06°04'36"S, 50°11'36"W), 1♀, 18/XI–01/XII/2010, L. Tunes (IBSP 164958); Cave N4WS_0015 (06°03'59"S, 50°11'22"W), 1♀, 20/X–01/XI/2006, R. Andrade et al. (IBSP 97741); Cave N4WS_0019 (06°04'35"S, 50°11'37"W), 1♀, 18/XI–01/XII/2010, L. Tunes col. (IBSP 164960), Cave N4WS_0021 (06°03'59"S, 50°11'24"W), 1♀, 18/XI–01/XII/2010, D. Bebiano (IBSP 164985); Cave N4WS_0024 (06°03'47"S, 50°11'29"W), 2♀, 18/XI–01/XII/2010, J. B. Verdiani (IBSP 164959); Cave N4WS_0029 (06°03'48"S, 50°11'28"W), 1 imm., 18/XI–01/XII/2010, L. Tunes (IBSP 164988); Cave N4WS_0033 (06°03'58"S, 50°11'23"W), 2♀, 18/XI–01/XII/2010, C.A.R. Souza (IBSP 164984); Cave N4WS_0050/51 (06°04'43"S, 50°11'34"W), 5♀ 1 imm., XI-XII/2010–09/VI/2011, D. Bebiano, C.A.R. Souza & I. Cizauskas col. (IBSP 164957, IBSP 164990, IBSP 164992); Cave N4WS_0054 (06°05'13"S, 50°11'40"W), 1♀, 18/XI–01/XII/2010, B. F. Takano (IBSP 164989), Cave N4WS_0057 (06°04'33"S, 50°11'28"W), 2♀ 1 imm., 10–19/V/2011, C.A.R. Souza (IBSP 164956); Cave N4WS_0066 (06°04’S 50°11'30"W), 3♂ 1♀ 1 imm., 18/XI‒01/XII/2010–10-19/V/2011, C.A.R. Souza & I. Cizauskas (IBSP 164954–164955); Cave N4WS_0073 (06°04'25"S, 50°11'37"W), 1♀, 18/XI-01/XII/2010, D. Bebiano (IBSP 164987); Cave N4WS_0074 (06°04'19"S, 50°11'22"W), 3♀, 18/XI–01/XII/2010, D. Bebiano (IBSP 164962); Cave N4WS_0077 (06°04'28"S, 50°11'18"W), 1♀, 10–19/V/2011, C.A.R. Souza col. (BSP 164963); Cave N4WS_0078 (06°04'20"S, 50°11'22"W), 4♀, 18/XI–01/XII/2010-10-19/V/2011, L. Tunes, D. Bebiano & F. P. Franco (IBSP 164961; IBSP 164986; IBSP 164991); Cave N5S_0005 (06°06'21"S, 50°08'01"W), 5♀, 4 imm., 14–23/X/2009, I. Cizauskas & J. B. Verdiani (IBSP 161038–161039; IBSP 161050); Cave N5S_0008 (06°06'21"S, 50°07'57"W), 3♀ 5 imm., 14‒23/X/2009, J. B. Verdiani & D. B. Pedroso (IBSP 161042, IBSP 161046); Cave N5S_0013 (06°06'19"S, 50°08'02"W), 2♀ 3 imm., 14‒23/X/2009, D. B. Pedroso (IBSP 191145); Cave N5S_0015 (06°06'20"S, 50°08’W), 13♀ 15 imm., 14-23/X/2009, R. Andrade, I. Cizauskas & J.B. Verdiani (IBSP 161033, IBSP 161035, IBSP 161044, IBSP 161048, IBSP 191146); Cave N5S_0017 (06°05'15"S, 50°07'11"W), 2♀ 1 imm., 25/VIII‒03/IX/2009, I. Cizauskas & D.B. Pedroso (IBSP 161045, IBSP 161047); Cave N5S_0022 (06°05'16"S, 50°07'33"W), 1♀ 2 imm., 25/VIII–03/IX/2009, J. Mascarenhas (IBSP 161034); Cave N5S_0026 (06°05'15"S, 50°07'38"W), 1♀, 10–19/V/2011, I. Cizauskas (IBSP 165000); Cave N5S_0058 (06°06'29"S, 50°07'57"W), 1♀, 14/III–04/IV/2010, J. B. Verdiani (IBSP 161041); Cave N5S_0059 (06°06'29"S, 50°07'57"W), 1♂ 1♀ 1 imm., 25/VIII-03/IX/2009, D. Bebiano (IBSP 161037); Cave N5S_0059 (06°06'29"S, 50°07'57"W), 1♂ 1♀, 25/VIII‒03/IX/2009, I. Cizauskas & J.B. Verdiani (IBSP 161040, IBSP 191158); Cave N5S_0063/64/65 (06°06'12"S, 50°08'07"W), 2♀ 1 imm., 15‒21/IX/2009, I. Cizauskas (IBSP 191144); Cave N5S_0067 (06°06'10"S, 50°08'07"W), 1♀ 2 imm., 14/III‒04/IV/2010, J. B. Verdiani (IBSP 161043); Cave N6_0005 (06°07'22"S, 50°10'28"W), 2♂, 16/VII-06/VIII/2014, Equipe Carste (IBSP 191188); Cave N8_0007 (06°10'05"S, 50°09'34"W), 1♂ 2♀, 16/VII‒06/VIII/2014, Equipe Carste (IBSP 191189‒191191); Cave N8_0019 (06°10'10"S, 50°09'25"W), 1♂ 1♀, 16/VII‒06/VIII/2014, Equipe Carste (IBSP 191192-191193); Canaã dos Carajás, Flona Carajás, Cave CAV_0039 (06°24'53"S, 50°22'23"W), 1♀ 3 imm., 22‒31/V/2010-22‒28/IX/2010, D. B. Pedroso, C.A.R. Souza & J. Mascarenhas (IBSP 164964‒164965, IBSP 191114); Cave GEM-1342 (06°16'02"S, 49°57'05"W), 1♀, 05‒15/III/2012 (IBSP 191208); Cave GEM-1475 (06°16'38"S, 49°55'05"W), 1♀ 1 imm., 17/I‒02/II/2012 (IBSP 191207); Cave GEM-1483 (06°16'35"S, 49°55'05"W), 1♀, 29/VIII‒27/IX/2012 (IBSP 191210); Cave GEM-1508 (06°18'55"S, 49°57'23"W),1♀, 29/VIII‒27/IX/2012 (IBSP 191209); Cave GEM-1517 (06°15'50"S, 49°58'38"W), 1♀, 10‒31/I/2013, (IBSP 191211), all collected by C.A.R. Souza & J. Mascarenhas et al.; Cave NV_0004 (06°28'42"S, 49°54'08"W), 1♀ 1 imm., 22–28/II/2005, R. Andrade & I. Amoni (IBSP 55364); Cave S11_0002 (06°26'21"S, 50°16'50"W), 1♂ 3♀ 4 imm., 24/II–19/VIII/2010, I. Cizauskas & V. Felice (IBSP 164993, IBSP 164995, IBSP 164997-164998); Cave S11A_0003 (06°21’S 50°27'02"W), 1 imm., 23/VIII–02/IX/2007, R. Andrade (IBSP 97825); Cave S11_0016 (06°25'10"S, 50°15'03"W), 3♂ 1♀ 2 imm., 24/II‒04/III/2010–19/VIII/2010, I. Cizauskas & D. Bebiano (IBSP 164994, IBSP 164996, IBSP 164999); Cave S11A_0020 (06°19'04"S, 50°26'23"W), 2♂ 1♀ 3 imm., 23/VIII-02/IX/2007, R. Andrade et al. (IBSP 97818); Cave S11B_0014 (06°20'59"S, 50°24'10"W), 1♀ 5 imm., 23/VIII–02/IX/2007, R. Andrade et al. (IBSP 97701); Cave S11B_0016 (06°20'53"S, 50°24'24"W), 1 imm., 23/VIII–02/IX/2007, R. Andrade et al. (IBSP 97968); Cave S11C_0020 (06°24'03"S, 50°22'50"W), 1♂ 2♀ 1 imm., 23/VIII‒02/IX/2007, R. Andrade et al. (IBSP 97687, IBSP 97863); Cave S11D_0003 (06°24'02"S, 50°21’W), 1♀, 01–14/VII/2010, I. Cizauskas (IBSP 164973); Cave S11D_0012 (06°23'46"S, 50°21'34"W), 1♀, 23/VIII‒02/IX/2007, R. Andrade et al. (IBSP 97801); Cave S11D_0049 (06°24'25"S, 50°19'14"W), 1♀, 13–30/I/2010, D. Bebiano (IBSP 164971); Cave S11D_0054 (06°24'22"S, 50°19'13"W), 2 imm., 13‒30/I/2010, R. Andrade (IBSP 164969); Cave S11D_0055 (06°24'23"S, 50°19'12"W), 2♂ 4♀, 01–14/VII/2010, I. Cizauskas (IBSP 164968, IBSP 191113); Cave S11D_0061 (06°23'33"S, 50°18'47"W), 1♂ 1♀ 1 imm., 13/I/2010–14/VII/2010, I. Cizauskas & J. Mascarenhas (IBSP 164967, IBSP 164975, IBSP 164977); Cave S11D_0064 (06°23'31"S, 50°18'48"W), 1♀, 23/VIII‒02/IX/2007, R. Andrade et al. (IBSP 97803); Cave S11D_0067 (06°23'34"S, 50°18'53"W), 1 imm., 13‒30/I/2010, J. B. Verdiani (IBSP 164981); Cave S11D_0072 (06°23'33"S, 50°19'09"W), 1 imm., 13‒30/I/2010, I. Cizauskas (IBSP 164974); Cave S11D_0076 (06°23'33"S, 50°19’W), 1♀ 1 imm., 13-30/I/2010, R. Andrade (IBSP 164982); Cave S11D_0079 (06°23'33"S, 50°18'56"W), 1♂, 01-14/VII/2010, V. Felice (IBSP 164970); Cave S11D_0083 (06°23'48"S, 50°19'25"W), 1♀, 13‒30/I/2010, I. Cizauskas (IBSP 164979); Cave S11D_0083 (06°23'48"S, 50°19'25"W), 3♂ 5♀, 23/VIII/2007–14/VII/2010, R. Andrade & I. Cizauskas et al. (IBSP 97793, IBSP 164976, IBSP 164978); Cave S11D_0085 (06°23'47"S, 50°19'24"W), 1 imm., 13‒30/I/2010, J. Mascarenhas (IBSP 164980); Cave S11D_0093 (06°23'41"S, 50°19'18"W), 1♀ 3 imm., 01-14/VII/2010, V. Felice & J. Mascarenhas (IBSP 164966, IBSP 164972); Cave S11D_0098 (06°23'46"S, 50°20'27"W), 1 imm., 03‒19/VIII/2010, D.B. Pedroso (IBSP 164983); Cave S11D_0107 (06°23'35"S, 50°18'45"W), 1♀, 30/VIII‒02/IX/2011, D. Bebiano (IBSP 191115); Cave SB-140 (06°21'05"S, 49°48'34"W), 1♀, 10‒31/I/2013 (IBSP 191213); Cave SB-149 (06°21'04"S, 49°50'30"W), 1♀ 3 imm., 10‒1/I/2013 (IBSP 191214); Cave SB-150 (06°21'04"S, 49°50'30"W), 2♀ 2 imm.,10‒31/I/2013 (IBSP 191212); Cave SB-210 (06°20'22"S, 49°57'36"W), 1♀, 10–20/IX/2013, (IBSP 19215), all collected by C.A.R. Souza & J. Mascarenhas et al.; Cave N5SM2_0001 (06°08'33"S, 50°08'03"W), 3♀ 14 imm. (ISLA); Cave N5SM2_0005 (06°08'28"S, 50°08'10"W), 3 imm. (ISLA); Cave N5SM2_0013 (06°08'18"S, 50°08'12"W), 1 imm. (ISLA); Cave N5SM2_0014 (06°08'20"S, 50°08'02"W), 2 imm. (ISLA); Cave N5SM2_0020 (06°08’S 50°07'53"W), 1 imm. (ISLA); Cave N5SM2_0021 (06°08’S 50°07'53"W), 2♀ 9 imm. (ISLA); Cave N5SM2_0022 (06°08'09"S, 50°08'09"W), 6 imm. (ISLA); Cave N5SM2_0023 (06°08'08"S, 50°08'07"W), 2♀ 3 imm. (ISLA); Cave N5SM2_0025 (06°08'10"S, 50°08'07"W), 1♀ 6 imm. (ISLA); Cave N5SM2_0026 (06°08'10"S, 50°08'08"W), 1 imm. (ISLA); Cave N5SM2_0027 (06°08'06"S, 50°08'12"W), 1 imm. (ISLA); Cave N5SM2_0029 (06°08'06"S, 50°08'11"W), 1♀ 10 imm. (ISLA); Cave N5SM2_0030 (06°08'05"S, 50°08'11"W), 4 imm. (ISLA); Cave N5SM2_0031 (06°08'04"S, 50°08'10"W), 2 imm. (ISLA); Cave N5SM2_0032 (06°08'04"S, 50°08'10"W), 1 imm. (ISLA); Cave N5SM2_0033 (06°08'04"S, 50°08'10"W), 1♀ 2 imm. (ISLA); Cave N5SM2_0034 (06°08'03"S, 50°08'10"W), 1♀ 7 imm. (ISLA); Cave N5SM2_0035 (06°08'03"S, 50°08'07"W), 3♀ 3 imm. (ISLA); Cave N5SM2_0037 (6°08’S 50°08'07"W), 1 imm. (ISLA); Cave N5SM2_0038 (06°07'59"S, 50°08'07"W), 2 imm. (ISLA); Cave N5SM2_0039 (06°07'59"S, 50°08'08"W), 2 imm. (ISLA); Cave N5SM2_0040 (06°08’S 50°08'13"W), 5 imm. (ISLA); Cave N5SM2_0041 (06° 08’S 50°08'14"W), 1 imm. (ISLA); Cave N5SM2_0043 (06°07'57"S, 50°08'12"W), 2♀ 5 imm. (ISLA); Cave N5SM2_0047 (06°07'54"S, 50°08'10"W), 1♀ 4 imm. (ISLA); Cave N5SM2_0050 (06°07'52"S, 50°08'07"W), 1♀ 2 imm. (ISLA); Cave N5SM2_0052 (06°07'52"S, 50°08'07"W), 2♀ (ISLA); Cave N5SM2_0055 (06°07'48"S, 50°08'06"W), 6♀ 12 imm. (ISLA); Cave N5SM2_0056 (06°07'48"S, 50°08'06"W), 1 imm. (ISLA); Cave N5SM2_0057 (06°07'48"S, 50°08'07"W), 1♀ 1 imm. (ISLA); Cave N5SM2_0059 (06°07'47"S, 50°08'07"W), 2♀ 2 imm. (ISLA); Cave N5SM2_0060 (06°07'45"S, 50°08'10"W), 1♂ 1♀ 7 imm. (ISLA); Cave N5SM2_0061 (06°07'44"S, 50°08'08"W), 3 imm. (ISLA); Cave N5SM2_0062 (06°07'43"S, 50°08'07"W), 7 imm. (ISLA); Cave N5SM2_0066 (06°07'42"S, 50°08'09"W), 1♀ 7 imm. (ISLA); Cave N5SM2_0067 (06°07'41"S, 50°08'14"W), 1 imm. (ISLA); Cave N5SM2_0070 (06°07'33"S, 50°07'56"W), 4 imm. (ISLA); Cave N5SM2_0071 (06°07'32"S, 50°07'56"W), 3 imm. (ISLA); Cave N5SM2_0072 (06°07'32"S, 50°07'56"W), 13 imm. (ISLA); Cave N5SM2_0073 (06°07'34"S, 50°07'57"W), 2 imm. (ISLA); Cave N5SM2_0074 (06°07'33"S, 50°07'57"W), 1♀ 1 imm. (ISLA); Cave N5SM2_0075 (06°07'33"S, 50°07'56"W), 9 imm. (ISLA); Cave N5SM2_0075 (06°07'33"S, 50°07'56"W), 4 imm. (ISLA); Cave N5SM2_0076 (06°07'32"S, 50°07'56"W); 1 imm. (ISLA); Cave N5SM2_0077 (06°07'30"S, 50°07'54"W), 2♀ 2 imm. (ISLA); Cave N5SM2_0078 (06°07'24"S, 50°07'50"W), 3 imm. (ISLA); Cave N5SM2_0078 (06°07'24"S, 50°07'50"W), 2 imm (ISLA); Cave N5SM2_0079 (06°07'24"S, 50°07'51"W), 4 imm. (ISLA); Cave N5SM2_0080 (06°07'21"S, 50°07'49"W), 7♀ 9 imm. (ISLA); Cave N5SM2_0081 (06°07'20"S, 50°07'46"W), 1♂ 5♀ 4 imm. (ISLA); Cave N5SM2_0082 (06°07'21"S, 50°07'44"W), 2♀ 11 imm. (ISLA); Cave N5SM2_0083 (06°07'22"S, 50°07'43"W), 5♀ 10 imm. (ISLA); Cave N5SM2_0084 (06°07'21"S, 50°07'42"W) 4 imm. (ISLA); Cave N5SM2_0086 (06°07'18"S, 50°07'49"W), 2 imm. (ISLA); Cave N5SM2_0090 (06°07'16"S, 50°07'47"W), 3♀ 7 imm. (ISLA); Cave N5SM2_0091 (06°07'16"S, 50°07'47"W), 10 imm. (ISLA); Cave N5SM2_0092 (06°07'19"S, 50°07'57"W), 1 imm. (ISLA); Cave N5SM2_0097 (06°07'43"S, 50°08'10"W), 5 imm. (ISLA); Cave N5SM2_0100 (06°07'19"S, 50°07'56"W), 1♀ 4 imm. (ISLA); Cave N5SM2_0101 (06°07'18"S, 50°07'56"W), 2 imm. (ISLA); Cave N5SM2_0102 (06°07'19"S, 50°07'54"W), 8♀ 20 imm (ISLA), 2007–2009, all collected by Equipe UFLA.

##### Etymology.

The specific name is a noun in apposition taken from the type locality.

##### Diagnosis.

Males of *Misionella carajas* can be distinguished from *Misionella aikewara* by the longer palpal tibia and shorter paraembolic lamina (Fig. 1K‒L: arrow; 2B‒D, 11A‒B) and from *Misionella pallida* by the narrow paraembolic lamina (Figs 1K‒L: arrow; 11A‒B). Females can be recognized by the shorter and not curved spermathecae (Fig. 11C).

##### Description.

Male (IBSP 161036). Carapace orange brown with lateral borders, thoracic groove and ocular area black. Sternum, chelicerae and labium orange. Legs and palp orange. Abdomen dorsally black and ventrally grayish (Figs 1A‒C, E‒F, 13B). Total length 4.1. Carapace 1.8 long, 1.3 wide. Eye diameters: PME 0.4, separated by about 2 diameters. Sternum with shallow longitudinal ventral sulcus, without sigillae. Palp: femur length 0.9, patella 0.4, tibia 1.1 long, 0.5 wide. Leg measurements: I: femur 3.8, patella 0.5, tibia 3.7, metatarsus 3.3, tarsus 1.8, total 13.1; II: 3.3, 0.5, 3.7, 2.4, 0.8, 10.7; III: 2.2, 0.4, 2.4, 2.6, 1.1, 8.7; IV: 3.1, 0.5, 3.0, 3.4, 1.4, 11.4. Metatarsus II with a pair of hirsute macrosetae (Figs 1G–I, 2A) on a cuticular outgrowth. Abdomen 2.2 long. Palp: tibia two times longer cymbium, with membranous area, cymbium short, and bulb globose and short, with elongated embolus (Figs 1K–L, 2B–D).

Female (IBSP 161040). Coloration as in male, but darker. Total length 5.6. Carapace 2.3, long, 1.5 wide. Serrula with 10‒11 teeth (Fig. 3D). Sternum as in male. Eye diameters: PME 0.5, separated by about 2 diameters. Pedipalp: length 1.4, patella 0.7, tibia 1.1, tarsus 0.4. Leg measurements: I: femur 3.1, patella 0.7, tibia 3.5, metatarsus 3.2, tarsus 1.5, total 12.0; II: 2.1, 0.7, 3.4, 2.0, 0.9, 9.1; III: 1.9, 0.6, 1.6, 1.7, 0.8, 6.6; IV: 2.6, 0.6, 2.4, 2.4, 1.0, 9.0. Legs with plumose hairs (Fig. 3C), trichobothria elevated and smooth (Fig. 3B), paired claws with 9 teeth and unpaired claw with two teeth (Fig. 3A) and calamistrum in three rows (Fig. 1D). Pedipalp hirsute (Fig. 1J) Abdomen 3.2 long. Posterior median spinnerets with one paracribellar gland spigot, along one minor ampullate gland and at least seven aciniform gland spigots (Fig. 3E). Posterior lateral spinnerets with large paracribellar gland spigots at the margin of spinning field and few aciniform gland spigots (Fig. 3F). Spermathecae with short ducts, close at base (Figs 11C, 12 A–D)

##### Variation.

10 males: total length 3‒4.2; carapace 1.4‒1.7; femur I 2.8‒4.5. 10 females: total length 4.2‒6.8; carapace 1.7‒2.4; femur I 2.7‒3.2.

##### Natural history.

This species is very common in the Carajás area, where 352 adult specimens were collected. 101 males, 251 females and approximately 400 immature (only 164 included here) were sampled in 144 caves, between the years 2006–2010. The caves are formed in iron ore in areas of residual plateau, more specifically on the bases of outcrops of iron ore or ‘canga’. The ‘canga’ are usually covered by open vegetation type called ‘metalophylic vegetation’, which is characterized by plants able to grow in soils rich in iron and other heavy metals (Pinto-da-Rocha and Andrade 2012; Carmo and Jacobi 2013). The specimens were found in lighter areas as well as in the darker areas of the interior of caves. They were always caught in the refuge of their irregular webs located on the ground and/or walls of the cavities (Figs 13A–C). Two types of refuges were found, one formed by tubes in the interior of the guano (Fig. 13C) and other, with irregular distribution of silk, in the walls or using the nest of wasps as substrate (Fig. 13D). The prey commonly observed were micro-Lepidoptera of the family Tineidae (Fig. 13A) and immature of Hemiptera of the genus *Zelurus* Silvestre (Reduviidae). Although the new species has been found only inside the caves, the specimens do not show any troglomorphism, except perhaps the elongate legs.

##### Distribution.

The species seems to occur exclusively in caves in the region of the Flona of Carajás, in the municipalities of Parauapebas and Canaã dos Carajás (Fig. 14).

#### Misionella aikewara sp. n.

Taxon classificationAnimaliaAraneaeFilistatidae

http://zoobank.org/FA3E70CA-B8BC-4C93-ABBE-060356DCB449

**Figure 4.**
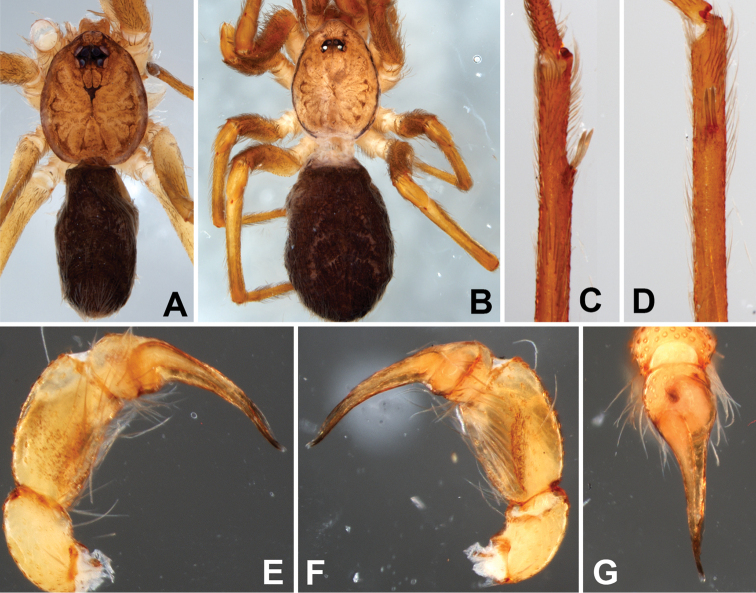
*Misionella aikewara* sp. n.. Male from São Geraldo do Araguaia, Pará (IBSP 191196) (**A, C–G**), female from Xambioá, Tocantins (IBSP 191194) (**B**). **A–B** habitus, dorsal view **C** male left metatarsus II, ventral view **D** male left metatarsus II, retrolateral **E** male left palp, prolateral view **F** same, retrolateral view **G** same, dorsal view.

Figs 4A–G , 11D–F , 13E , 14

##### Type material.

Male holotype from Cave SI-07 (788310 9295476), São Geraldo do Araguaia, Pará, Brazil, 31.VIII–09.IX.2009, F. P. Franco et al., deposited in IBSP 191196; female paratype from Cave SI-04 (786471 9290451), Xambioá, Tocantins, 31.VIII–-09.IX.2010, F. P. Franco et al., deposited in IBSP 191194.

##### Additional material examined.

BRAZIL. *Pará*: São Geraldo do Araguaia, Cave SI-30 (783442 9304748), 1♀, 31.VIII‒09.IX.2010, F. P. Franco et al. (IBSP 191197); 2 imm., 22.II‒02.III.2011, F. P. Franco et al. (IBSP 191199); *Tocantins*: Ananás, Cave SI-13 (785816 9310724), 1♀, 31.VIII‒09.IX.2010, F. P. Franco et al. (IBSP 191195); Cave SI-13, 2♀, 27‒31.I.2011 (IBSP 191198); *Tocantins*: Miracema do Tocantins (09°34'02"S, 48°23'30"W), 3♀ 1 imm., 17‒25.IV.2005, I. Knysak & R. Martins, in a cave at night (IBSP 124517).

##### Etymology.

The specific name is a noun in apposition and refers to the ethnic group of the region of São Geraldo do Araguaia, where the type locality is located: the Tupi indigenous group Aikewará.

##### Diagnosis.

Males of *Misionella aikewara* can be distinguished from *Misionella carajas* and *Misionella pallida* by the shorter palpal tibia and elongated paraembolic lamina (Figs 4E–G, 11D‒E). Females can be recognized by the elongated receptacles curved distally and separated at the base (Fig. 11F).

##### Description.

Male (holotype). Carapace orange with brown submarginal bands. Thoracic groove and ocular area black. Chelicerae orange. Labium and endites yellowish. Sternum yellowish with brown borders. Legs and palps orange. Abdomen dark brown (Fig. 4A). Total length 2.7. Carapace 1.2, long, 1.0 wide. Sternum with small and shallow sulcus, without sigillae. Eye diameters: PME 0.4, separated by your diameters. Palp: femur length 2.0, patella 1.0, tibia 1.2 long, 0.8 wide. Leg measurements: I: femur 2.8, patella 0.5, tibia 1.9, metatarsus 1.8, tarsus 0.9, total 7.9; II: 2.0, 0.4, 2.1, 1.3, 0.6, 6.4; III: 1.3, 0.5, 1.3, 1.3, 0.7, 5.1; IV: 2.0, 0.5, 1.9, 1.8, 0.9, 7.0. Metatarsus II with a pair of hirsute macrosetae (Fig. 4C–D). Abdomen 1.5 long. Palp: tibia shorter, two times the length of cymbium, bulb globose (Fig. 4E–G).

*Female* (IBSP 191194, cave SI-04). Coloration pattern as in male (Fig. 4B), except endites orange and legs darker. Total length 5.6. Carapace 2.1 long, 1.6 wide. Sternum as in male. Eye diameters: PME 0.4, separated by 2 diameters. Palp: femur length 1.5, patella 0.7, tibia 0.8, tarsus 0.9. Leg measurements: I: femur 2.9, patella 0.9, tibia 3.0, metatarsus 2.8, tarsus 1.2, total 10.8; II: 2.1, 0.7, 2.0, 1.8, 0.9, 7.5; III: 1.8, 0.7, 1.4, 1.5, 0.8, 6.2; IV: 2.0, 0.8, 2.1, 2.0, 0.9, 7.8. Abdomen 3.2 long. Spermathecae with elongated ducts and curved apex (Fig. 11F).

##### Variation.

5 females: total length 3‒4.5; carapace 1.4‒2; femur I 1.7‒2.2.

##### Natural history.

Eleven specimens were collected, only one male, eight females and two immature, in four limestone caves located in municipalities very close to the border of the states of Pará and Tocantins (Fig. 14). In general, the walls of the caves had high humidity with pools and/or running water therein. These caves have high number of micro-habitats such as roots, guano and crevices. The specimens of *Misionella aikewara* sp. n. were located in lighter areas as well as in the darker areas of the interior of caves. The webs are irregular, as in *Misionella carajas* sp. n., and the capture was always performed in the refuge of their webs located on the walls and cracks in the cavity (Fig. 13E). All specimens were found inside caves and do not show any kind of troglomorphism.

##### Distribution.

This species occurs only in the region of the State Park of Serra das Andorinhas, in states of Pará and Tocantins (Fig. 14).

#### Misionella pallida sp. n.

Taxon classificationAnimaliaAraneaeFilistatidae

http://zoobank.org/39C0C8CD-8EF0-4507-96E4-CC643E479536

Figs 5A–H , 6A–D , 7A–E , 8A–D , 9A–F , 10A–F , 11G–I , 12B–D , 13E , 14

##### Type material.

Male holotype from Bairro Morada do Sol (5°3'56"S, 42°46'1,02"W), Teresina, Piauí, Brazil, 30.I.2006, L.S. Carvalho col., deposited in MPEG 22760. Paratypes: two females from Parque Nacional de Sete Cidades (4°5'39,9"S, 41°43'53,3"W), Brasileira/Piracura, Piauí, Brazil, 3.XII.2006, L.S. Carvalho, D. Candiani & N.F.L. Man Hung col., deposited in MPEG 22748; male and female from Parque Municipal Pedra do Castelo (5°12'5,9"S, 41°41'14,2"W), Castelo do Piauí, Piauí, Brazil, 9.V.2004, L.S. Carvalho et al. col. deposited in CHNUFPI 604 and 605, respectively; male from Bairro Morada do Sol, Teresina, Piauí, Brazil (into a house), 5°3'56"S, 42°46'1"W, 15.X.2015, L.S. Carvalho col. (CHNUFPI 1624); male and female from Mossoró (5°11'16"S, 37°20'38"W), Rio Grande do Norte, Brazil, 29.X.2007, I.T. Rocha & D. Araujo col. (IBSP 91662, IBSP 91663).

##### Additional material examined.

BRAZIL. *Piauí*: José de Freitas, Fazenda Nazareth (4°45'21"S, 42°34'33"W), 1♂ 1♀ 2 imm, 12.X.2003, J. Riceti (MPEG 22747); Francinópolis, Sítio Vigário (6°23'46"S, 42°15'44.82"W), 1♀, 7.IV.2007, E.B.O Marques (MPEG 22749); Oeiras (7°01'30"S, 42°07'51"W), 1♂ 2 imm, 3.VI.2008, Yamaguti (MPEG 22755; MPEG 22751); Teresina, Campus UFPI, CCA (5°3'56"S, 42°46’W), 1♂, 2.III.2007, L.S. Carvalho (MPEG 22753); Teresina, Bairro São Joaquim, 7° DP (5°3'56"S, 42°46"W), 2♂, 22.I.2007, S.C. Carvalho (1 ♂, SEM; MPEG 22756); Teresina, Bairro Morada do Sol (5°3'56"S, 42°46'1,02"W), 1♀ 1 imm., 11.VII.2007, L.S. Carvalho (MPEG 22746); 1♂ 1♀, 4.X.2013, L.S. Carvalho (CHNUFPI 601); 1♂, I.2014, L.S. Carvalho (UFMG 14827; UFMG 14829); Brasileira/Piracura, Parque Nacional Sete Cidades (4°5'39,9"S, 41°43'53,3"W), 1 imm., 25.VI.2007 (MPEG 22757); 1♀, 03.XII.2006 (MPEG 22752); 1 imm., 31.I.2007 (MPEG 22761); 1 imm., 31.I.2007 (MPEG 22754), all collected by L.S. Carvalho, D. Candiani & N.F. Lo Man Hung; Coronel José Dias, Parque Nacional da Serra da Capivara (08°53'07"S, 42°33'12"W), 1♀, 07/VI/2012, L.S. Carvalho (UFMG 14829); Castelo do Piauí, Fazenda Bonito, E.C.B. Rochas Ornamentais (5°14'7,5"S, 41°41'16,3"W), 2♂, 13.VIII.2008, L.S. Carvalho (MPEG 22745; MPEG 22759); Castelo do Piauí, Parque Municipal Pedra do Castelo (5°12'5,9"S, 41°41'14,2"W), 2♂ 11♂ 1 imm., 9.V.2003, L.S. Carvalho et al. (UFMG 14384-14394; UFMG 14387; CHNUFPI 602; CHNUFPI 603); União, Sítio Ouro Verde (04°54'14"S, 42°47'21"W), 1♂, 25/V/2014, L.S. Carvalho (CHNUFPI 1099); Floriano, campus da UFPI (06°47'29"S, 43°2'50"W), 4/IX/2013, L.S. Carvalho, 1♂ (UFMG 14828). *Maranhão*: Caxias, Reserva Ecológica do Inhamum (04°53'30"S, 43°24'53"W), 30♂, 23‒26.IV.2007, F.B. Lima-Lobato (IBSP 129097; IBSP 131101; IBSP 131029; IBSP 129092; IBSP 129088; IBSP 131024; IBSP 129093; IBSP 129095; IBSP 130967; IBSP 131022; IBSP 99121; IBSP 98671; IBSP 131027; IBSP 131025; IBSP 129090, IBSP 98670); *Rio Grande do Norte*: Mossoró (5°11'16"S, 37°20'38"W), 1♀, 29.X.2008, I.T. Rocha & D. Araujo (IBSP 91661). *Bahia*: Brumado, Magnesita (14°12'14"S, 41°39'55"W), 1♀, E.A. Araújo, 02‒3/V/2014 (UFMG 15513).

##### Etymology.

The name is an adjective referring to the pale coloration of the body in both males and females of this species.

##### Diagnosis.

**Figure 5.**
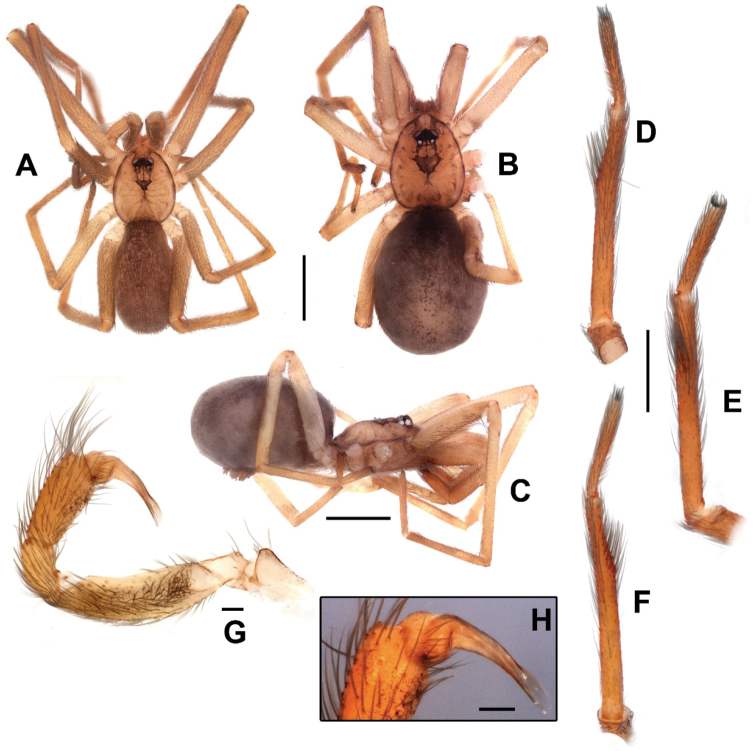
*Misionella pallida* sp. n.. **A, G–H** Male from Bairro Morada do Sol, Teresina, Piauí (UFMG 14827) **D–F** Sítio Ouro Verde, União, Piauí (CHNUFPI 1099) **B–C** female from Parque Nacional Serra da Capivara, Coronel José Dias, Piauí (UFMG 14829). **A–B** habitus, dorsal view **C** habitus, lateral view **D** male right leg II, ventral view **E** same, retrolateral view **F** same, dorsal view **G** male right palp, prolateral view **H** same, distal area (mirrored). Scale bars: 1 mm (**A–C**), 0.5 mm (**D–F**), 0.1 mm (**G–H**).

**Figure 6.**
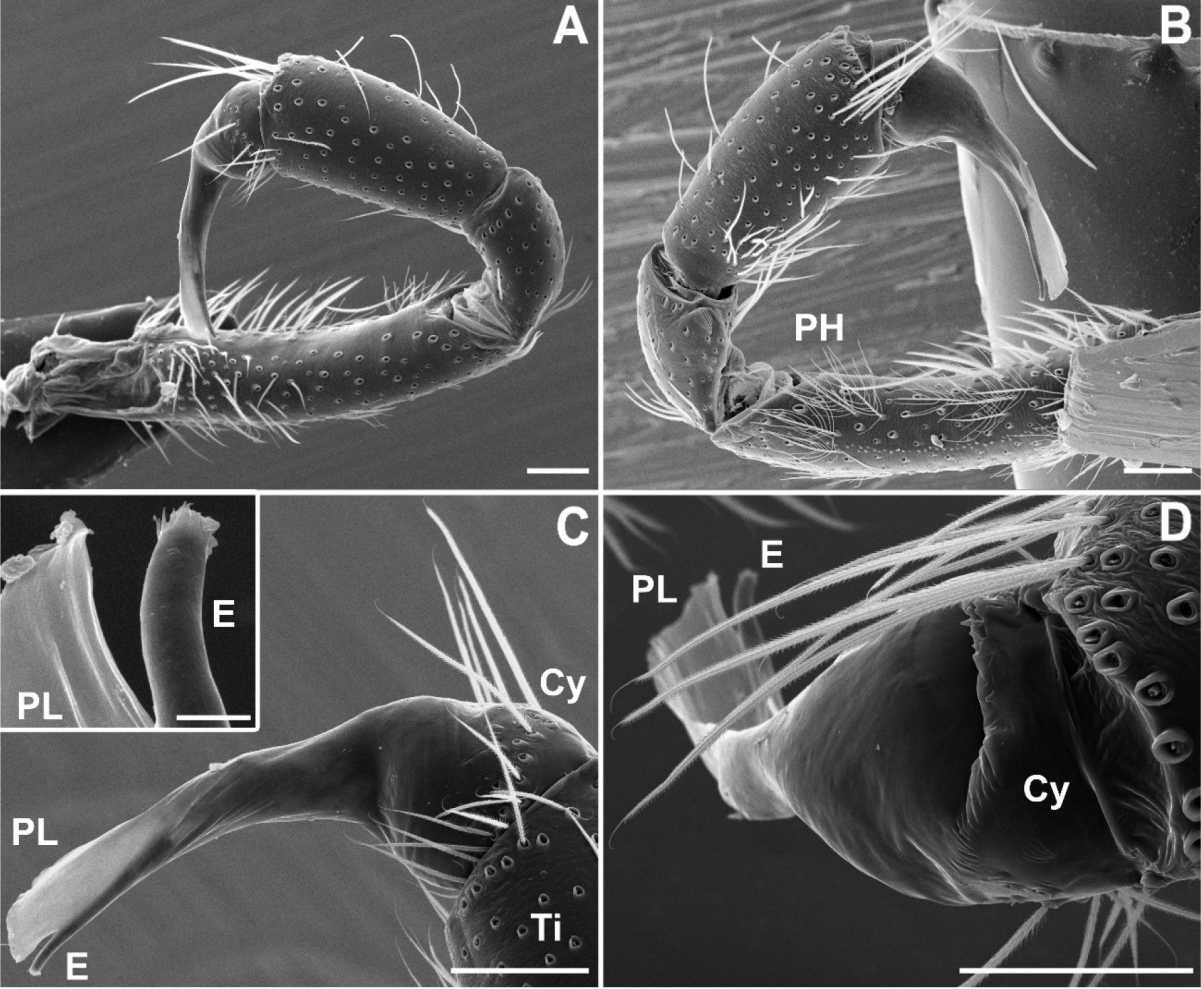
SEM images of *Misionella pallida* sp. n., male from Floriano, Piauí, Brasil (UFMG 14828) **A** male right palp, prolateral view **B** same, retrolateral view **C** same, prolateral view, detail of right bulb (inset embolus and PL, dorsal) **D** same, right cymbium, dorsal view. Abbreviations: Cy = cymbium, E = embolus, PH = plumose setae, PL = paraembolic lamina, Ti = palpal tibia. Scale bars: 0.1 mm, except inset, 0.01 mm.

*Misionella pallida* can be distinguished from other *Misionella* species by the pale coloration of the body. Males are further distinguished by the large and flattened paraembolic lamina (Figs 5G–H, 6A–E: PL, 11G–H). Females can be recognized by the long, distally incrassate, and largely separated distal area of the spermathecae (Fig. 11I).

##### Description.

**Figure 7.**
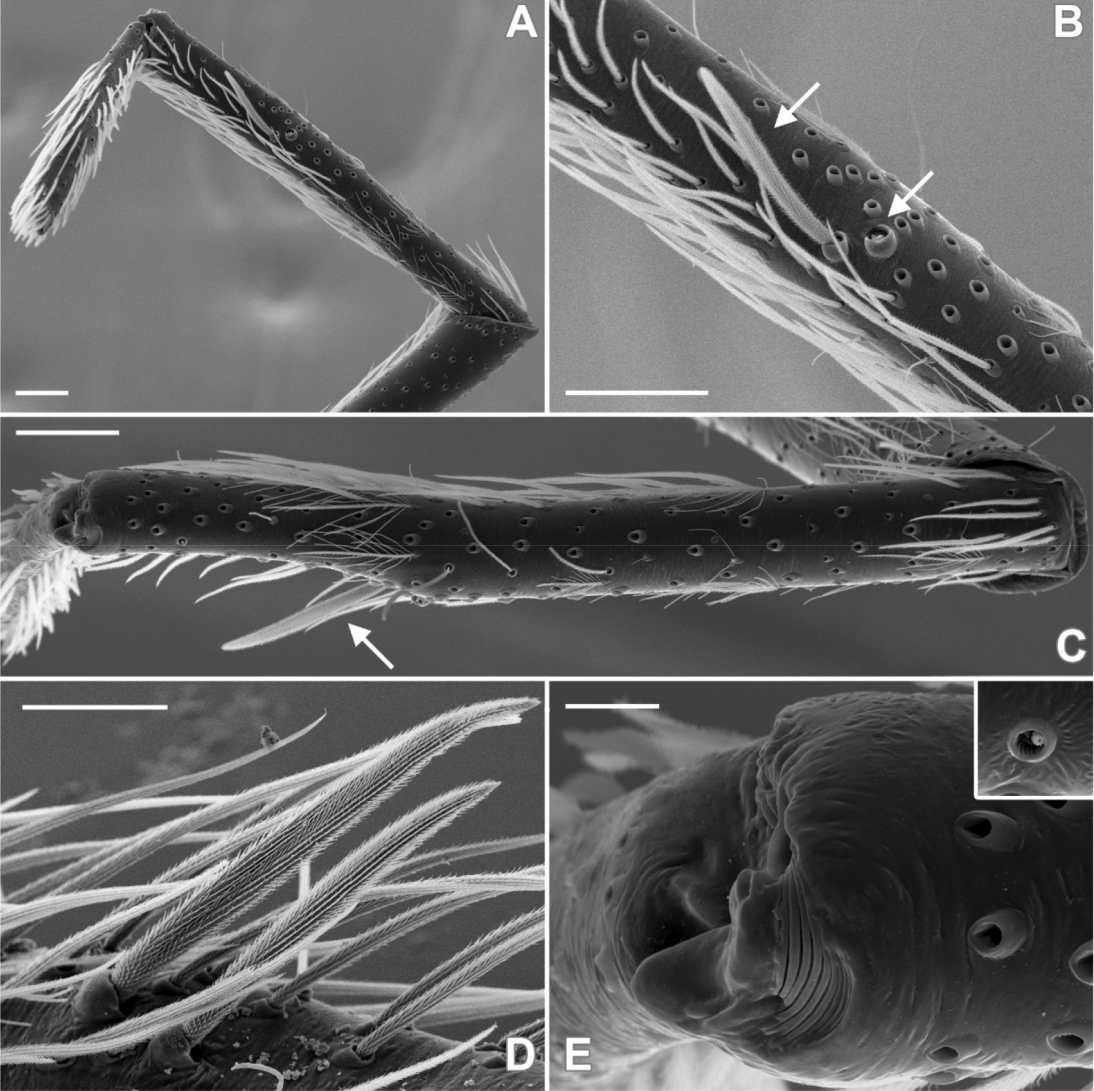
SEM images of *Misionella pallida* sp. n., male from Bairro Morada do Sol, Teresina, Piauí (UFMG 14827), **A** left leg II, retrolateral view **B** same, detail of macrosetae, retrolateral view. Arrow points to spines. **C** same, dorsal view. Arrow points to macrosetae **D** same, detail of macrosetae, subventral view **E** same, metatarsus stopper, dorsal view (inset, tricobothrial base). Scale bars: 0.1 mm (**A–C**), 0.05 mm (**D**), 0.02 mm (**E**).

Male (MPEG 22756). Carapace orange with thoracic groove, lateral stripes and border black. Ocular area black. Chelicerae and labium orange. Endites and sternum cream. Legs and palps orange. Abdomen dorsally greyish, with grey stripes in the anterior border, and ventrally cream (Fig. 5A). Total length 3.0. Carapace 1.3 long, 1.0 wide. Sternum with small and shallow sulcus, without sigillae. Eye diameters: PME 0.4, separated by about four diameters. Pedipalp: femur length 0.9, patella 0.3, tibia 0.5 long, 0.3 wide. Leg measurements: I: femur 2.5, patella 1.0, tibia 3.0, metatarsus 2.3, tarsus 1.1, total 9.9; II: 1.8, 0.5, 2.0, 1.2, 0.5, 6.0; III: 1.3, 0.4, 1.0, 1.1, 0.4, 4.2; IV: 1.9, 1.0, 2.0, 1.7, 0.6, 7.2. Metatarsus II with a pair of hirsute macrosetae (Figs 5D–F, 7A–D). Abdomen 1.7 long, epiandrous area with at least 15 fusules (Fig. 9A). Cribellum divided and smooth (Fig. 9B). Spinnerets: ALS with one major ampullate gland spigot and at least 20 piriform gland spigots, PMS with one minor ampullate gland spigot, two aciniform gland spigots and one elongated paracribellar gland spigot, PLS with one paracribellar gland spigot and two aciniform gland spigots (Fig. 9B, D–F). Palp: tibia elongated, short cimbyum, bulb globose, with large and flattened paraembolic lamina (Figs 5G–H, 6A–E, 7A–E, 11G–H).

**Figure 8.**
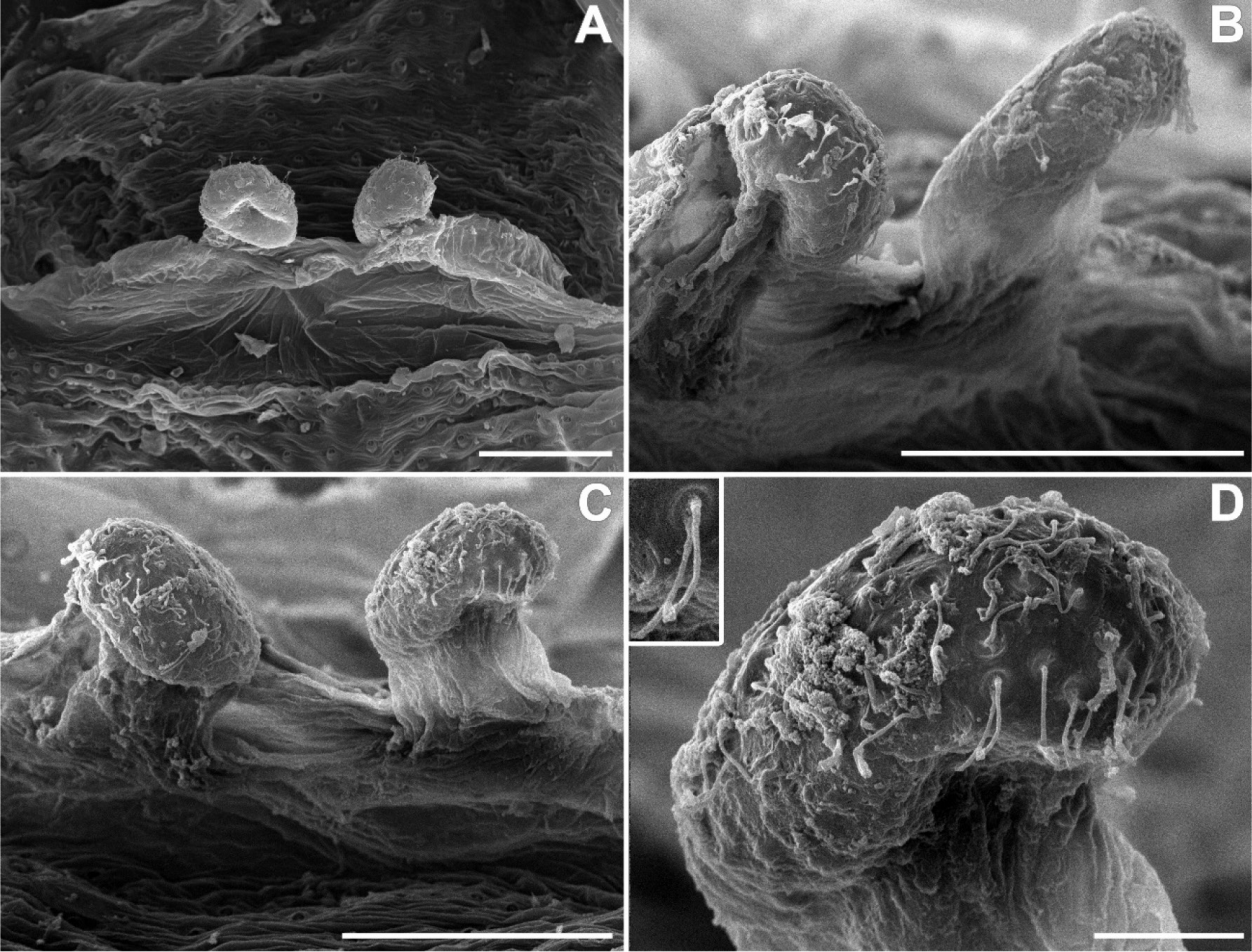
SEM images of *Misionella pallida* sp. n., female from Parque Municipal Pedra do Castelo, Castelo do Piauí, Piauí (UFMG 14385) **A** spermathecae, dorsal view **B** same, lateroventral view **C** same, anteroventral view **D** same, anteroventral (inset: detail of pores with filamentous gland). Scale bars: 0.1 mm (**A–C**), 0.02 mm (**D**).

**Figure 9.**
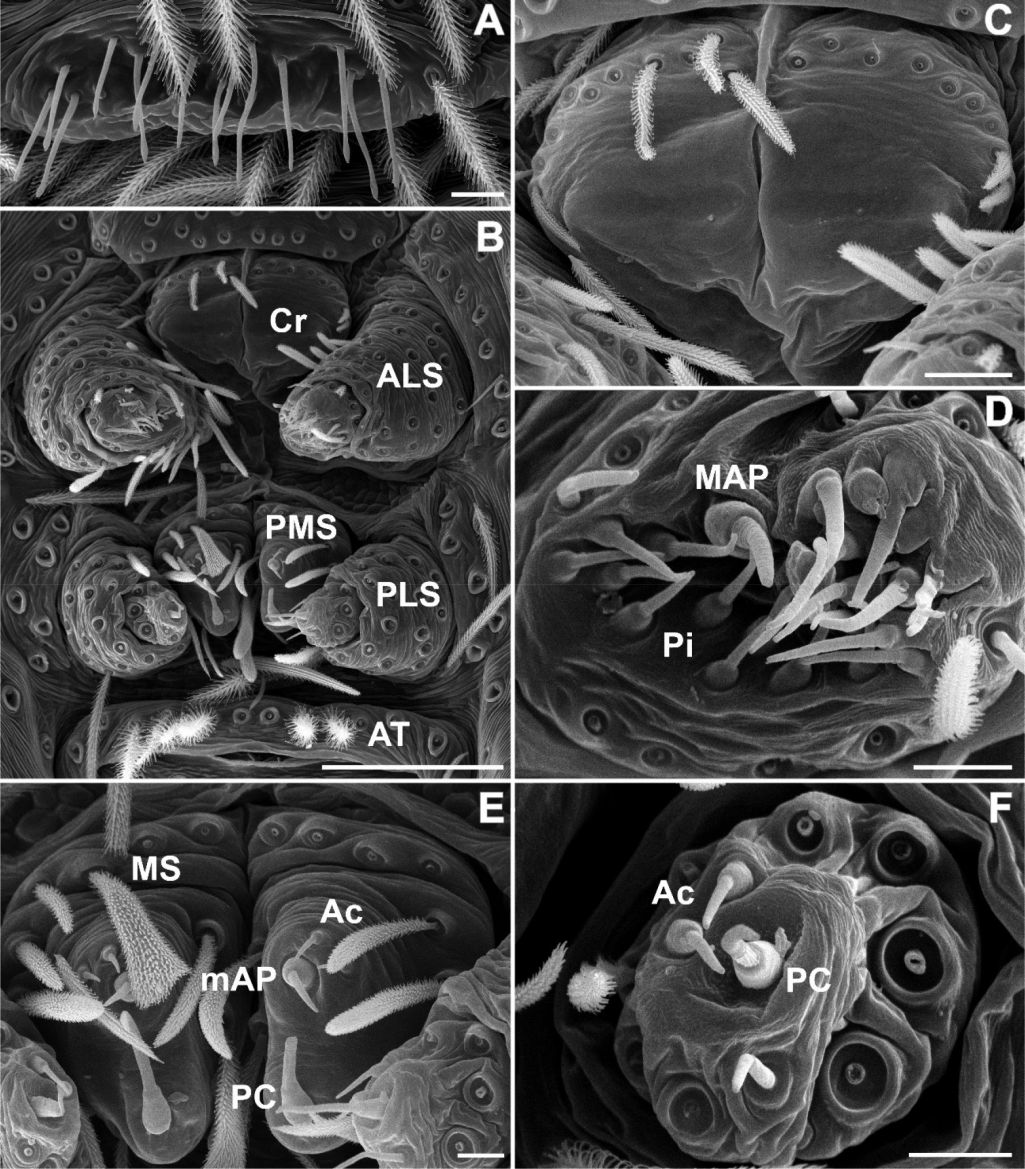
SEM images of *Misionella pallida* sp. n., male from Floriano, Piauí, Brasil (UFMG 14828) **A** epiandrium, ventral view **B** spinnerets, ventral view **C** cribellum, ventral view **D** spinnerets, left ALS, ventral view **E** PMS, ventral view **F** left PLS, ventral view. Abbreviations: Ac = aciniform gland spigots, ALS = anterior lateral spinnerets, AT = anal tubercle, Cr = cribellum, MAP = major ampullate gland spigot, mAP = minor ampullate gland spigot, PC = paracribellar gland spigot, Pi = piriform gland spigot, PLS = posterior lateral spinnerets, PMS = posterior median spinnerets. Scale bars: 0.02 mm (**A, C**), 0.1 mm (**B**), 0.01 mm (**D–F**).

**Figure 10.**
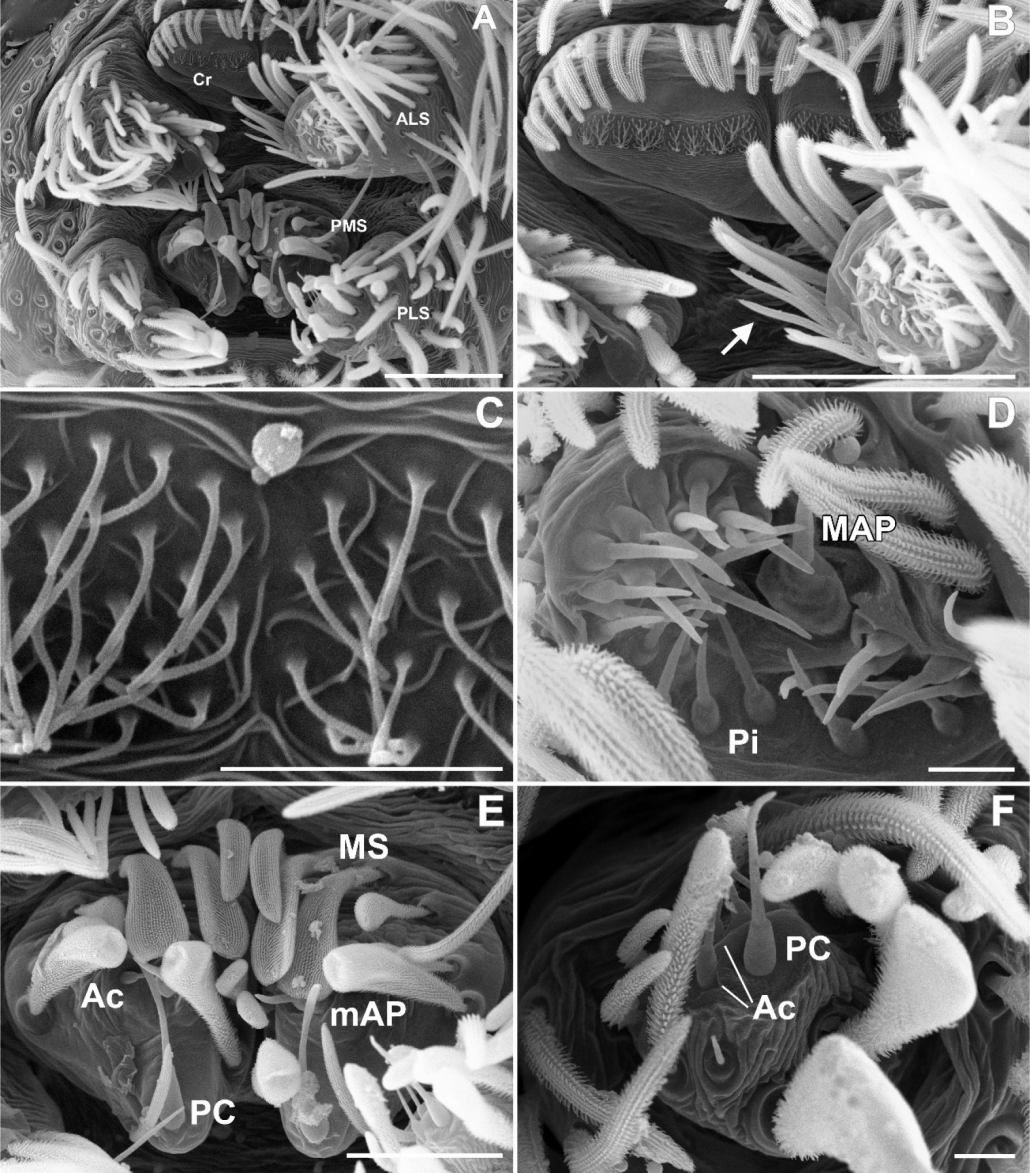
SEM images of *Misionella pallida* sp. n., female from Parque Municipal Pedra do Castelo, Castelo do Piauí, Piauí (UFMG 14385) **A** spinnerets, ventral view **B** same, cribellum and left ALS, ventral view. Arrow points to row of setae on ALS, characteristic of filistatids **C** same, cribellar spigots, ventral view **D** same, right ALS, ventral view **E** PMS, ventral view **F** right PLS, ventral view. Abbreviations: Ac = aciniform gland spigots, ALS = anterior lateral spinnerets, Cr = cribellum, MAP = major ampullate gland spigot, mAP = minor ampullate gland spigot, PC = paracribellar gland spigot, Pi = piriform gland spigot, PLS = posterior lateral spinnerets, PMS = posterior median spinnerets. Scale bars: 0.02 mm (**A–B**), 0.01 mm (**C–D, F**), 0.05 mm (**E**).

**Figure 11.**
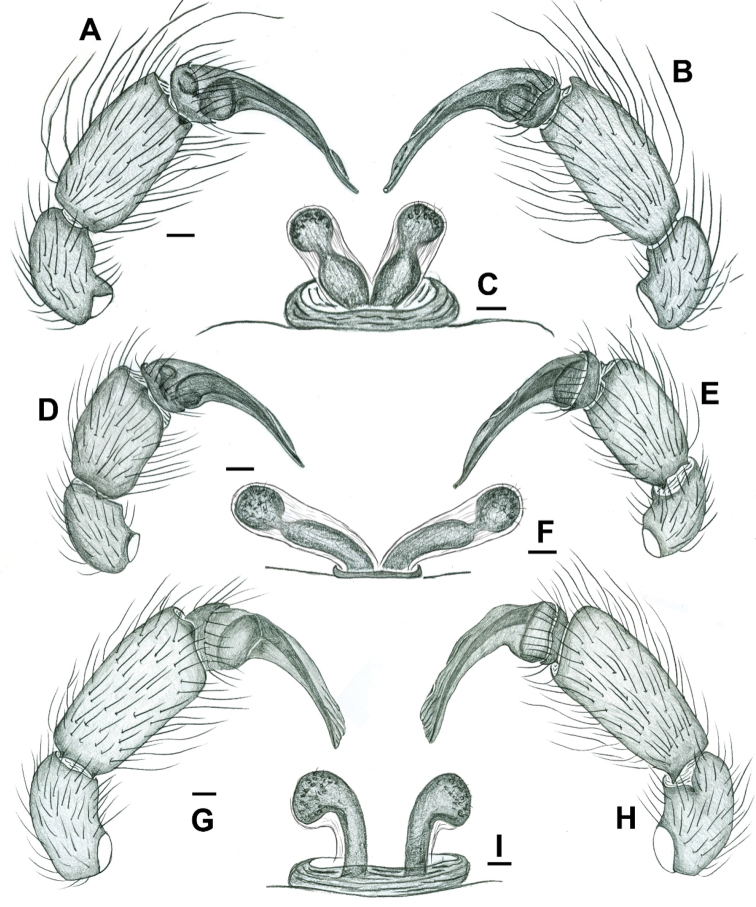
*Misionella carajas* sp. n., male and female from Cave N5S_0059, Flona Carajás, Parauapebas, Pará (IBSP 161040) (**A–C**) *Misionella aikewara* sp. n., male from São Geraldo do Araguaia, Pará (IBSP 191196) (**E**), female from Xambioá, Tocantins (IBSP 191194) (**F**). *Misionella pallida* sp. n., male and female from Bairro São Joaquim, Teresina, Piauí (MPEG 22756) (**G–I**). **A–B** male palp. **A** prolateral view **B** retrolateral view **C** spermathecae, dorsal view **D–E** male palp, prolateral view **E** retrolateral view **F** spermathecae, dorsal view **G–H** male palp **G** prolateral view **H** retrolateral view **I** spermathecae, dorsal view. Scale bars: 0.1 mm (**A–B, D–E, G–H**), 0.02 mm (**C, F, I**).

*Female* (MPEG 22748). Coloration as in male, except pedipalp red brown and abdomen greenish gray with anterior dorsal border black (Fig. 5B–C). Total length 4.0. Carapace 1.7 long, 1.2 wide. Sternum as in male. Eye diameters: PME 0.4, separated by about 2 diameters. Pedipalp: femur length 1.1, patella 0.5, tibia 0.7, tarsus 0.3. Leg measurements: I: femur 1.8, patella 0.5, tibia 2.1, metatarsus 1.4, tarsus 1.0, total 6.8; II: 1.4, 0.5, 1.2, 1.0, 0.6, 4.7; III: 1.2, 0.4, 0.9, 1.0, 0.5, 4.0; IV: 1.7, 0.5, 1.5, 1.3, 0.6, 5.6. Abdomen 2.4 long. Cribellum as in male, but more numerous spigots (Fig. 10B–C). Spinnerets: ALS as in male, PMS with one minor ampullate gland spigot, at least one aciniform gland spigots and one elongated paracribellar gland spigot, PLS with one paracribellar gland spigot and four aciniform gland spigots (Fig. 10A, D–F). Spermathecae curved at tip with elongated ducts (Figs 8A–D, 11I).

**Figure 12.**
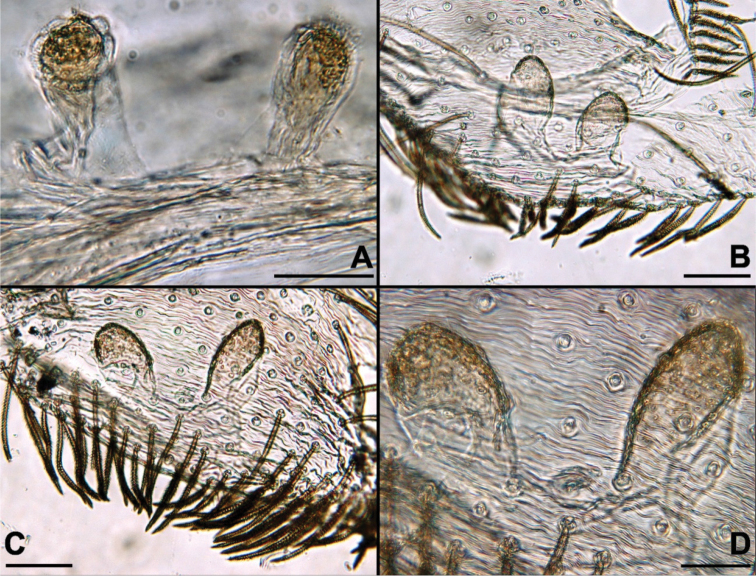
Spermathecae, dorsal view, latic acid cleared. **A** *Misionella carajas* sp. n., female from Cave N4E_0079, Flona Carajás, Parauapebas, Pará (IBSP 166200) **B** *Misionella pallida* sp. n., female from Parque Nacional das Sete Cidades, Piracuruca, Piauí (MPEG 22752) **C–D** same species, variation, female from Magnesita, Brumado, Bahia (UFMG 15513). Scale bars: 0.05 mm (**A, D**), 0.1 mm (**B–C**).

**Figure 13.**
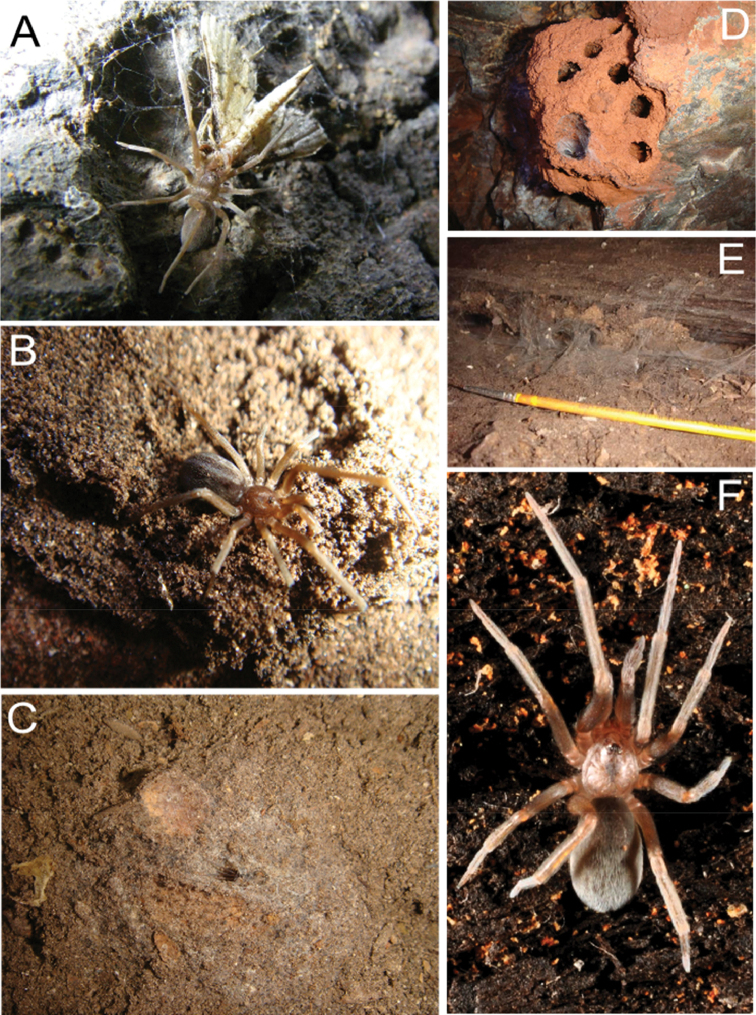
*Misionella carajas* sp. n. (**A–D**), **A** capturing a Tineidae (Lepidoptera) **B** male, dorsal view **C** soil web in bat guano **D** female living in nests of wasps (Hymenoptera); *Misionella aikewara* sp. n. **E** refuge formed the web in the wall of cave **F** *Misionella pallida* sp. n., female, dorsal view.

##### Variation.

10♂: total length 1.9‒2.8; carapace 1‒1.3; femur I 1.4‒2.6; 10♀: total length 3.8‒4.2; carapace 1.6‒1.8; femur I 1.8‒2.1.

##### Natural history.

This species has been collected several times in both natural and synanthropic habitats in northeastern Brazil. The species seems to naturally occur in Caatinga vegetation, a type of seasonally dry tropical forest. In synanthropic conditions, females can be found in their webs in the corners and cracks of windows and doors (L.S. Carvalho, pers. comm.). Males have been collected in pitfall traps in Caxias, in the state of Maranhão.

##### Distribution.

**Figure 14.**
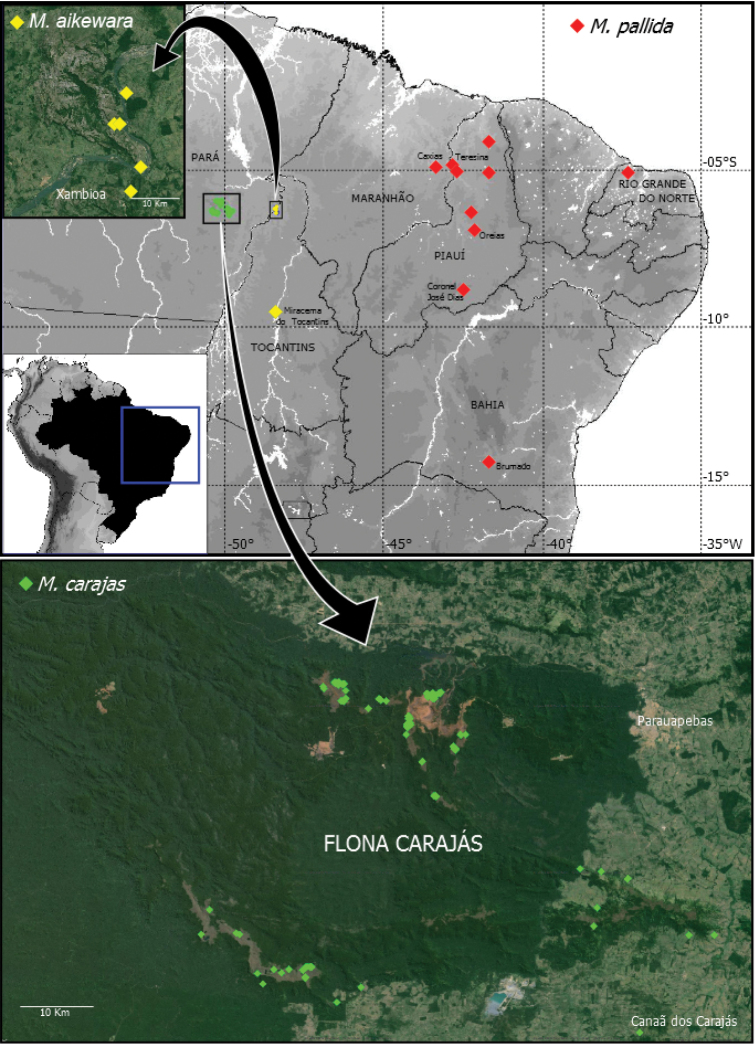
Map showing records of the three new *Misionella* species in Brazil. Green diamond = *Misionella carajas* sp. n., yellow diamond = *Misionella aikewara* sp. n., red diamond = *Misionella pallida* sp. n.

Known from Northeastern Brazil (Fig. 14).

## Discussion

**Phylogenetic placement.** The three new species herein described superficially resemble *Filistatoides* F.O. Pickard-Cambridge due to the elongate palps and bulbs, and by the female genitalia with a single pair of spermathecae (see Gray 1995; Ramírez and Grismado 1997). However, they share two derived states with the South American genera *Pikelinia* and *Misionella*: the cymbium fused to the tegulum (Fig. 6C, D) and the second metatarsus of males modified and bearing retrolateral macrosetae (Figs 1G–I, 2A, 4C–D, 5D–F, 7). Currently, *Misionella* is defined by the presence of a modified metatarsus II combined with the absence of a tibial apophysis in the male palp (Ramírez and Grismado 1997), both characters being present in the three new species. However, several undescribed filistatid species not treated here have intermediate morphologies between *Misionella* and *Pikelinia* (Magalhaes, unpublished data), blurring the limits between the two genera. The three species newly described apparently form a monophyletic group, supported by the unilobulate spermathecae, long bulbs, and the absence of pigment rings in the legs. Their placement in *Misionella* is not satisfactory, and a new genus could be proposed. However, as stressed above, the limits between South American filistatid genera are currently somewhat dubious, and several genus-defining characters are apparently homoplastic. A new phylogenetic analysis of the Filistatidae is in progress by the second author, and we think it is more prudent not to erect a new genus at the moment.

**Biogeography.** Filistatids are known to occur mainly in arid and semi-arid environments. To date, *Misionella* have been an exception as they seem to prefer more humid habits: *Misionella mendensis* occurs in the Cerrado (a savannah) and the Atlantic Forest, and *Misionella jaminawa* is Amazonian (Ramírez and Grismado 1997; Grismado and Ramírez 2000). On the other hand, *Misionella pallida* sp. n. seems to be restricted to the western border of the Caatinga, a seasonally dry tropical forest with a semi-arid climate (Pennington et al. 2000). *Misionella carajas* sp. n. and *Misionella aikewara* sp. n. occur in the humid Amazon, but they are restricted to caves. Caves often have different micro-climatic conditions than the surroundings, and sometimes harbor relict species (*e.g.* Malek-Hosseini et al. 2015). It has been hypothesized that the limits of the seasonally dry tropical forests of Brazil have changed in response to recent climatic fluctuations (Pennington et al. 2000; Magalhaes et al. 2014). Thus, it can be hypothesized that the ancestors of *Misionella carajas* sp. n. and *Misionella aikewara* sp. n. also lived in dry conditions, and that they reached their current distribution during an event of expansion of the dry forests, being subsequently ‘trapped’ in the caves as the dry forests receded. A dated phylogeny of South American prithine would help shed some light on these questions.

## Supplementary Material

XML Treatment for Misionella

XML Treatment for Misionella carajas

XML Treatment for Misionella aikewara

XML Treatment for Misionella pallida
